# Expression of Human Beta-Defensins in Children with Chronic Inflammatory Bowel Disease

**DOI:** 10.1371/journal.pone.0015389

**Published:** 2010-10-22

**Authors:** Matthias Zilbauer, Andreas Jenke, Gundula Wenzel, Jan Postberg, Andreas Heusch, Alan D. Phillips, Gabriele Noble-Jamieson, Franco Torrente, Camilla Salvestrini, Robert Heuschkel, Stefan Wirth

**Affiliations:** 1 Department of Paediatrics, HELIOS Klinikum Wuppertal, Germany; 2 Department of Paediatric Gastroenterology, Addenbrooke's Hospital, Cambridge, United Kingdom; 3 Centre for Paediatric Gastroenterology, Royal Free Hospital, London, United Kingdom; Charité-Universitätsmedizin Berlin, Germany

## Abstract

**Background:**

Human beta-defensins (hBDs) are antimicrobial peptides known to play a major role in intestinal innate host defence. Altered mucosal expression of hBDs has been suggested to be implicated in chronic inflammatory bowel disease pathogenesis. However, little is known about expression of these peptides in children.

**Methods:**

Intestinal biopsies were obtained from the duodenum (n = 88), terminal ileum (n = 90) and ascending colon (n = 105) of children with Crohn's disease (n = 26), ulcerative colitis (n = 11) and healthy controls (n = 16). Quantitative real-time (RT) PCR was performed and absolute mRNA copy numbers analyzed for hBD1-3 as well as inflammatory cytokines IL-8 and TNF-alpha.

**Results:**

Significant induction of hBD2 and hBD3 was observed in the inflamed terminal ileum and ascending colon of IBD children. In the ascending colon induction of hBD2 was found to be significantly lower in children with Crohn's disease compared to ulcerative colitis. A strong correlation was found between inducible defensins hBD2 and 3 and the inflammatory cytokines IL-8 and TNF-alpha, both in the terminal ileum and ascending colon.

**Conclusion:**

Our study demonstrates distinct changes in hBD expression throughout the intestinal tract of children with IBD, lending further support for their potential role in disease pathogenesis.

## Introduction

Human beta-defensins (hBDs) are a group of evolutionarily conserved antimicrobial peptides (AMPs) known to play a major role in innate host defence at various mucosal surfaces including the gastrointestinal (GI) tract. Three of the most extensively studied members are hBD1, -2 and -3. Under physiological conditions, hBD1 is expressed constitutively by intestinal epithelial cells while hBD2 and -3 are induced during infection and inflammation [1], [2]. In addition to their potent antimicrobial properties against commensal and pathogenic bacteria [3], [4], beta-defensins have been shown to function as multieffector molecules capable of enhancing host defence by recruiting various innate as well as adaptive immune cells to the site of infection, induction of neovasculogenesis and enhancing wound closure [5], [6], [7]. Given these versatile functions it is not surprising that impaired expression and/or function of these peptides has been implicated in the development of several diseases, including chronic inflammatory bowel disease (IBD). The two major entities are Crohn's disease (CD) and ulcerative colitis (UC), which differ in disease distribution as well as histological changes of the affected bowel segment. While CD causes transmural inflammation throughout the entire GI mucosa, changes in UC are restricted to the colonic mucosa. IBD disease pathogenesis is highly complex and remains incompletely understood. However, increasing evidence suggests that impaired or altered innate host defence mechanism(s) are likely to play a major role as highlighted by the association of NOD2 mutations and CD [8].

Several studies have reported on modulated hBD expression in the mucosa of IBD patients. For example, both constitutively expressed hBD1 as well as inducible defensins hBD-2 and -3 were found to be markedly up-regulated in the inflamed colonic mucosa of adults with IBD [9], [10], [11]. Moreover, induction seemed to be less pronounced in CD compared to UC [9]. In addition to mucosal hBD expression, reduced gene copy numbers for hBD1 and hBD2 have been shown to predispose to the development of CD - further highlighting the likely involvement of these peptides in IBD disease pathogenesis [12], [13]. However, studies published to date have been performed almost exclusively on adult patients with longstanding disease, and very little is known about hBD expression in paediatric IBD.

The aim of this study was to investigate expression of hBD1-3 in small and large bowel biopsies of children with IBD and healthy controls. Furthermore, the potential impact of mucosal inflammation on hBD expression was analysed by assessing histological changes and mucosal expression of the inflammatory cytokines IL-8 and TNF-alpha.

## Results

### Intestinal human beta-defensin expression in healthy children

**Figure 1:**
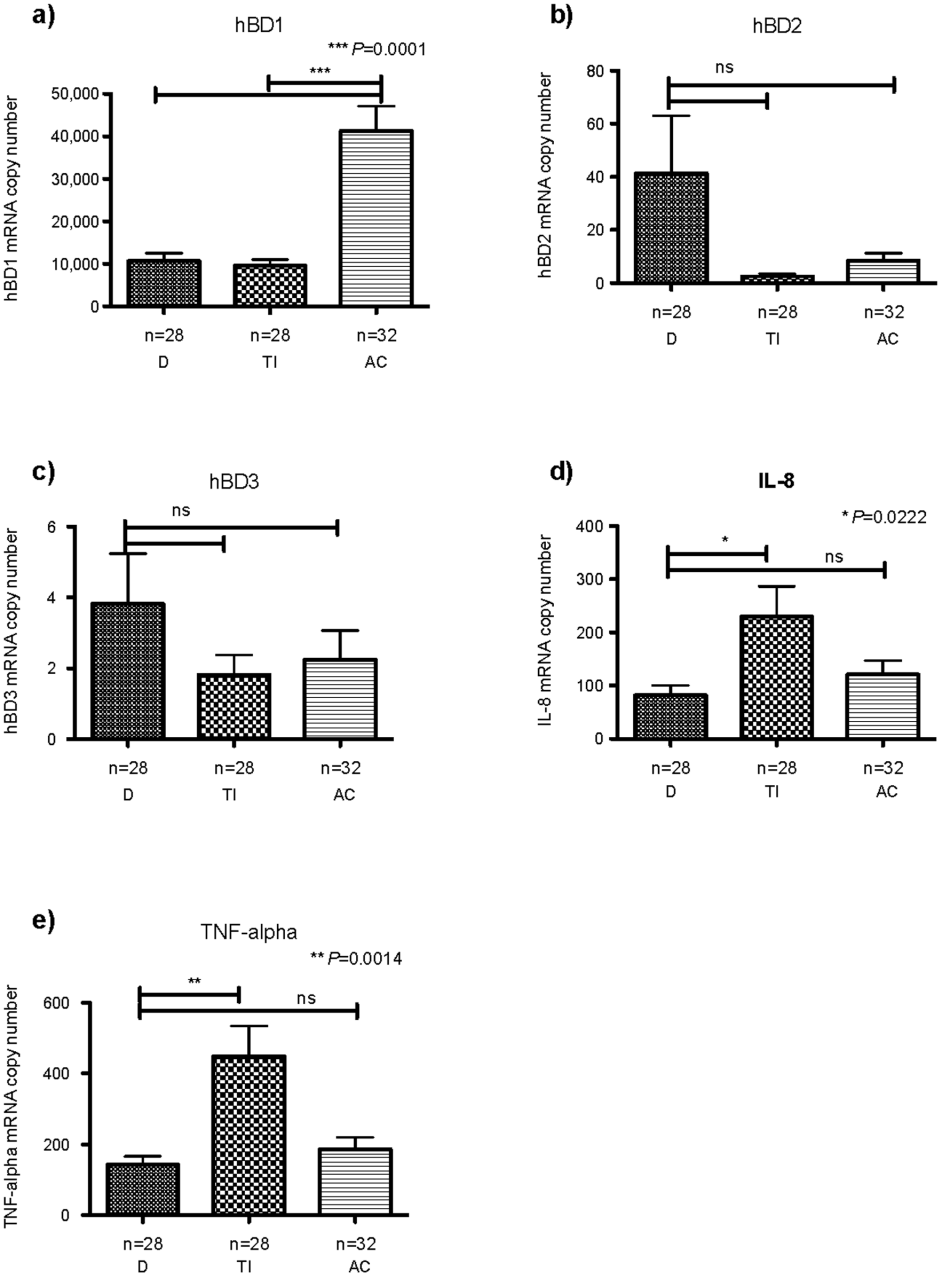
Expression of hBDs and inflammatory cytokines in the intestinal mucosa of healthy children. Biopsies were obtained from duodenum (D), terminal ileum (TI) and ascending colon (AC). Absolute mRNA copy number for hBD1-3 (a-c), IL-8 (d) and TNF-alpha (e) in each biopsy were quantified by real-time PCR using the standard curve method. Expression levels were normalized against the geometrical mean of two selected housekeeping genes (GPDH and β-actin). Data is expressed as the mean ± standard error of mean (SEM). The number of biopsies analysed for each patient group is stated (n). Values for *P*<0.05 were considered to be statistically significant.

First we analysed expression of hBD1-3 throughout the intestinal tract of healthy children. hBD1 was expressed constitutively in small and large bowel biopsies with levels being significantly higher in the colon compared to duodenum and TI (Figure 1a). In contrast to hBD1, inducible defensins hBD2 and -3 were infrequently expressed, with low copy numbers and no significant differences between bowel segments (Figure 1a and b). In addition to beta-defensins we also analysed mucosal expression of inflammatory cytokines IL-8 and TNF-alpha. As shown in Figure 1d and e, significantly higher levels for both IL-8 (P = 0.0222) and TNF-alpha (P = 0.0014) were found in the healthy TI compared to duodenum and ascending colon.

### Intestinal hBD1 expression in children with IBD and healthy controls

**Figure 2:**
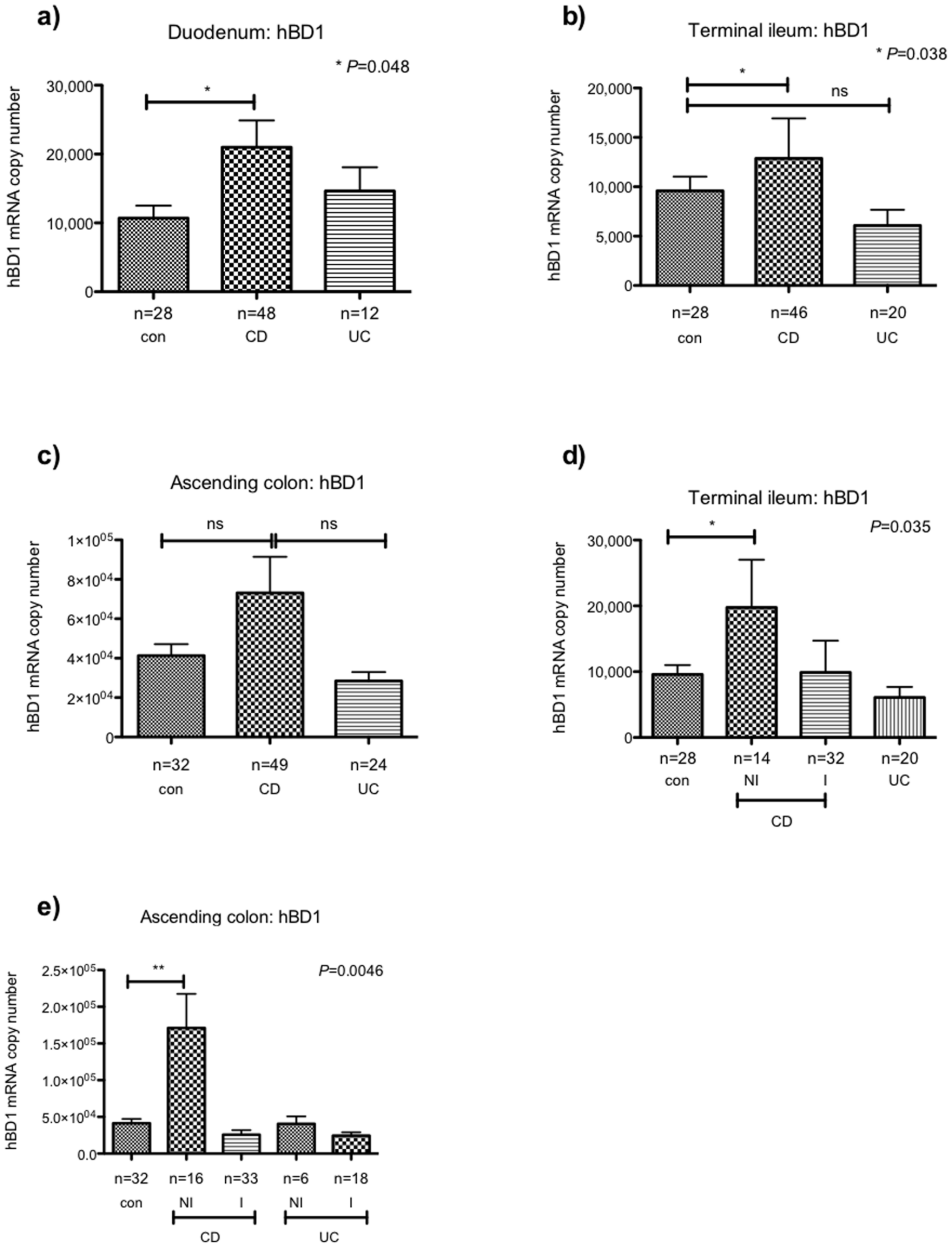
Expression of hBD1 in mucosal biopsies from IBD children and healthy controls. Expression of hBD1 in the duodenum (a), terminal ileum (b) and ascending colon (c) was analysed in children with Crohn's disease (CD), ulcerative colitis (UC) and healthy controls (con). For the TI (d) and AC (e), biopsy samples from CD patients were subdivided into histologically non-inflamed (NI) and inflamed (I). Data is expressed as the mean ± standard error of mean (SEM) of absolute mRNA copy numbers. The number of biopsies analysed for each patient group is stated (n). Values for *P*<0.05 were considered to be statistically significant.

Next we investigated expression of hBD1 in our patient cohort. Comparing hBD1 levels in children with UC and healthy controls, no difference was found in any of the bowel segments tested (Figure 2a–c). In contrast, significantly higher mRNA copy numbers were observed in duodenal and TI biopsies obtained from CD children compared to controls. Despite higher hBD1 levels in colonic biopsies of CD patients, this did not reach statistical significance (Figure 1c). However, subdividing IBD biopsies further into histologically inflamed (I) and non-inflamed (NI), revealed significantly higher hBD1 levels in the non-inflamed ascending colon and TI (Figure 2d and e).

### Expression of inducible beta-defensins and inflammatory cytokines in paediatric small bowel biopsies

**Figure 3:**
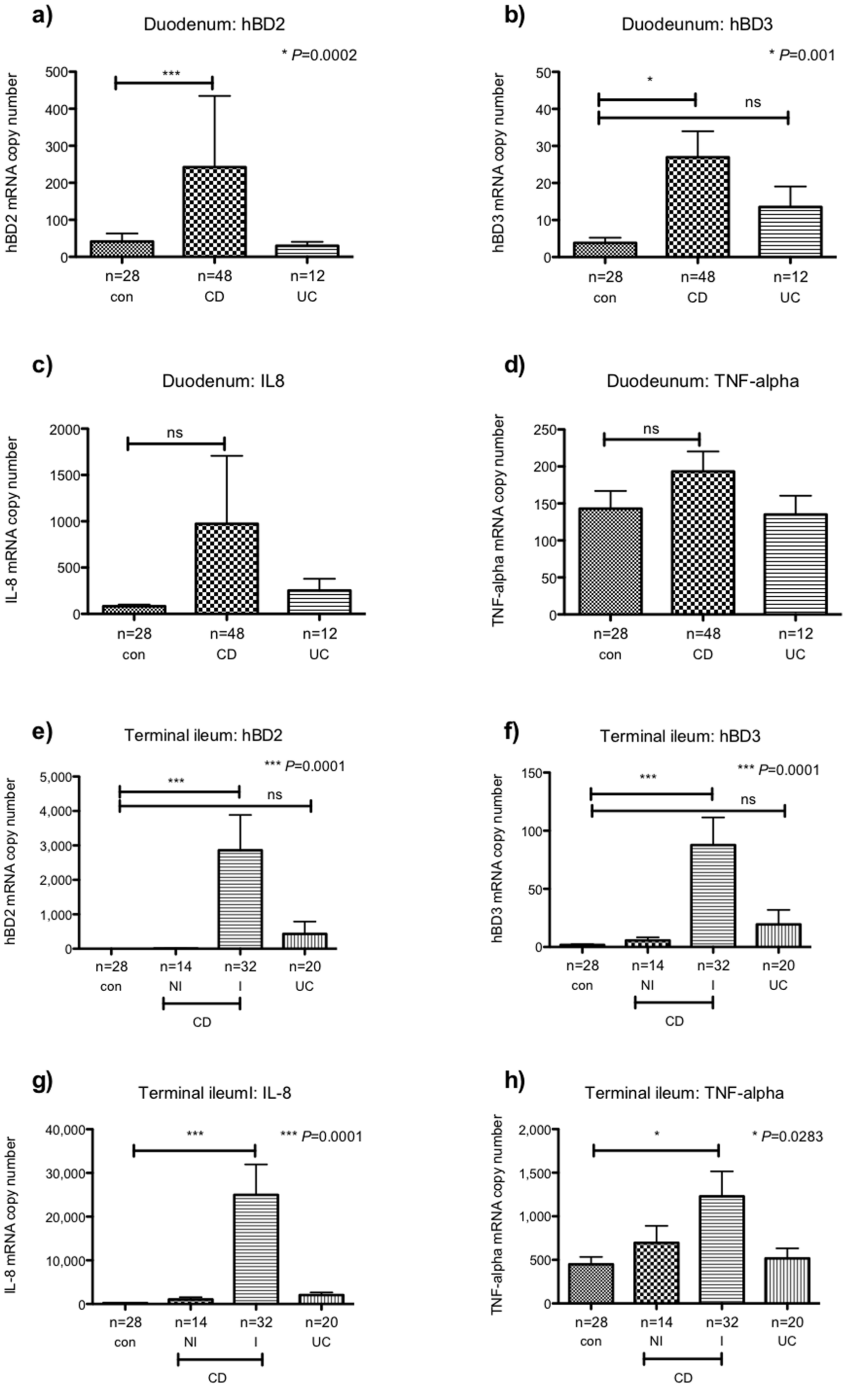
Expression of inducible beta defensins and inflammatory cytokines in small bowel biopsies of IBD children and healthy controls. Expression of hBD2 (a and e), hBD3 (b and f), IL-8 (c and g) and TNF-alpha (d and h) was analysed in the duodenum (a–d) and TI (e–h) of children with Crohn's disease (CD), ulcerative colitis (UC) and healthy controls (con). For the TI, biopsy samples from CD patients were subdivided into histologically non-inflamed (NI) and inflamed (I). Data is expressed as the mean ± standard error of mean (SEM) of absolute mRNA copy numbers. The number of biopsies analysed for each patient group is stated (n). Values for *P*<0.05 were considered to be statistically significant.

As shown in Figure 1 expression levels of hBD2 and hBD3 are low in the healthy mucosa. However, up-regulation has been shown to occur during infection and inflammation. We therefore analysed expression of hBD2 and -3 as well as inflammatory cytokines IL-8 and TNF-alpha in small bowel biopsies of our patient cohort. As shown in Figure 3a-d, significant up-regulation of both inducible beta-defensins was found in the duodenum of CD children. However, interestingly, this was despite the absence of significant inflammation as assessed by histological examination and expression of inflammatory cytokines IL-8 and TNF-alpha (Figure 3c and d). In contrast to the duodenum, significantly increased levels of both hBD2 and hBD3 were found exclusively in the inflamed mucosa of CD patients, which also expressed high levels of IL-8 and TNF-alpha.

### Colonic expression of inducible beta-defensins and inflammatory cytokines in paediatric IBD

**Figure 4:**
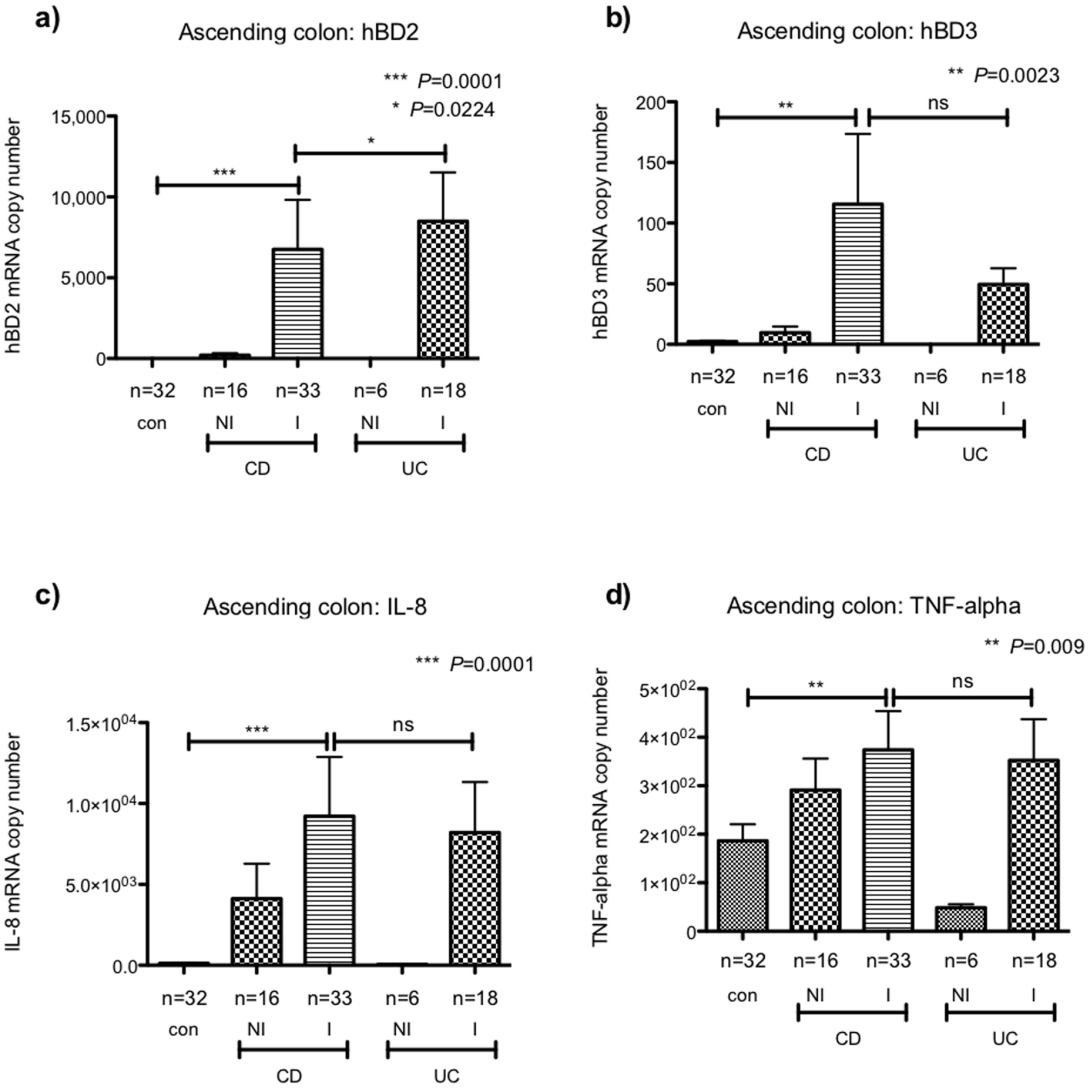
Expression of inducible beta defensins and inflammatory cytokines in colonic biopsies of children with IBD and healthy controls. Expression of hBD2 (a), hBD3 (b), IL-8 (c) and TNF-alpha (d) was analysed in biopsies obtained from the ascending colon of children with Crohn's disease (CD), ulcerative colitis (UC) and healthy controls (con). Biopsies from CD and UC patients were subdivided into histologically non-inflamed (NI) and inflamed (I). Data is expressed as the mean ± standard error of mean (SEM) of absolute mRNA copy numbers. The number of biopsies analysed for each patient group is stated (n). Values for *P*<0.05 were considered to be statistically significant.

In addition to the small bowel, we also analysed beta-defensin expression in colonic biopsies of IBD patients. Similar to our findings in the TI, expression of hBD2 and -3 was significantly induced in the inflamed mucosa of children with CD and UC (Figure 4a and b). Interestingly, induction of hBD2 was found to be significantly less pronounced in CD compared to UC biopsies despite equal levels of inflammatory cytokines IL-8 and TNF-alpha (Figure 4c and d). In contrast to hBD2, no difference was found in hBD3 levels comparing inflamed colonic mucosa of both patient groups. Significant induction of IL-8 and TNF-alpha in the histologically inflamed (I) colonic mucosa was observed. Moreover, increased levels of both inflammatory cytokines were also noted in histologically non-inflamed (NI) colonic biopsies of CD children (Figure 4c and d).

### Correlation of intestinal beta-defensin and inflammatory cytokine expression

Having noted significant changes of both constitutively and inducible beta-defensins in the inflamed GI mucosa if children with IBD, we analysed the correlation between inducible defensins hBD2 and hBD3 and the inflammatory cytokines within each bowel segment. For these calculations, both results of all children with IBD (inflamed and non-infloamed) and those of healthy control biopsies were included. As shown in Table 1, a highly significant correlation of hBD2 and hBD3 with IL-8 as well as TNF-alpha was found in TI and AC biopsies. In contrast, correlation analysis in the non-inflamed duodenum were largely non significant.

**Table 1:** Correlation of intestinal beta-defensin and inflammatory cytokine mRNA expression levels.

**Duodenum (n = 88)**	**TNF-alpha**	**IL-8**
**hBD2**	ns	*
	P = 0.389	P = 0.047
	*r* = 0.09295	*r* = 0.2124
**hBD3**	ns	ns
	P = 0.1982	P = 0.2295
	*r* = −0.1385	*r* = 0.129
**Terminal Ileum (n = 94)**	**TNF-alpha**	**IL-8**
**hBD2**	*	***
	P = 0.0143	P<0.0001
	*r* = 0.2519	*r* = 0.6101
**hBD3**	*	***
	P = 0.0269	P<0.0001
	*r* = 0.2283	*r* = 0.5951
**Ascending Colon (n = 105)**	**TNF-alpha**	**IL-8**
**hBD2**	***	***
	P<0.0001	P<0.0001
	*r* = 0.393	*r* = 0.6556
**hBD3**	**	***
	P = 0.0011	P<0.0001
	*r* = 0.3145	*r* = 0.5738

Correlation of gene expression between beta-defensins (hBD2 and hBD3) and inflammatory cytokines (IL-8 and TNF-alpha) in the duodenum, terminal ileum and ascending colon was tested by calculating Spearman's rank correlation coefficient (*r*). The number of biopsies included into calculations is stated (n). Values for *P*<0.05 were considered to be statistically significant.

## Discussion

Human beta-defensins have been shown to play a key role in intestinal innate host defence [14]. It is therefore not surprising that altered expression and/or function of these peptides has been implicated in IBD disease pathogenesis. However, little is known about the role and regulation of hBDs in paediatric patients. In this study we have investigated beta-defensin gene expression in over 250 intestinal biopsies obtained from children with IBD and healthy controls.

First we analysed expression of hBD1-3 and the inflammatory cytokines TNF-alpha and IL-8 in the mucosa of healthy children. We were able to confirm previously reported data showing constitutive expression of hBD1 throughout the GI tract while hBD2 and -3 were either not detected or present in low mRNA copy numbers [1]. Following this we investigated hBD expression in children with IBD. Here we observed significant induction of hBD2 and hBD3 in the inflamed TI and AC of children with CD as well as UC. Moreover, hBD2 induction in the inflamed colonic mucosa of CD children was found to be significantly less prominent compared to UC despite equal levels of IL-8 and TNF-alpha. This data is consistent with previously published findings on adult populations [9], [15], however it seems that hBD2 expression is less impaired in paediatric compared to adult CD, perhaps suggesting that further regulation occurs in long standing disease. In contrast to hBD2, no significant difference in TI or colonic hBD3 induction was observed between patient groups. This is again in contrast to previously published data on adult patients [9].

With regards to expression of hBD1, we found mRNA levels to be increased in the small and large bowel of CD patients only, whereas levels in UC biopsies did not differ from healthy control samples, further highlighting distinct differences between CD and UC. Interestingly, sub-dividing biopsies into inflamed and non-inflamed samples revealed that hBD1 up-regulation was mainly found in the non-inflamed mucosa of CD children. This however has to be interpreted with caution since it could simply signify a degree of epithelial cell loss within the inflamed tissue.

Given the fact that changes of hBD expression were particularly prominent in the inflamed small and large bowel, we analysed correlation of hBDs with inflammatory cytokines IL-8 and TNF-alpha. As expected, we found a strong positive correlation between the inducible defensins hBD2 and hBD3 and both inflammatory cytokines in the TI and AC confirming previous reports [16]. In contrast, analysis in the non-inflamed duodenum proved to be largely non-significant, suggesting regulatory mechanisms may vary between bowel segments.

Overall our data suggests differential regulation of hBDs in the mucosa of children with IBD, depending both on disease type and region of the gut. Specifically, induction of hBD2 and hBD3 in the inflamed TI and AC mucosa is likely to be of biological relevance for IBD disease pathogenesis. However, the exact mechanism(s) involved remain to be elucidated. Despite the observed reduction in hBD2 induction in CD compared to UC, one should be cautious to conclude that an impaired expression of hBDs is a main causative feature of CD. Importantly, in addition to the well-documented antimicrobial properties of hBDs, evidence is rapidly increasing on their role as potent immune modulators capable of enhancing inflammatory processes and hence potentially contributing to the onset and/or persistence of chronic intestinal inflammation [7]. Functional studies are required to shed further light on these important questions.

## Materials and Methods

### Patient cohort and sample collection

Patients for this study were recruited over a period of 18 months (08/2008 to 02/2010) from 3 paediatric gastroenterology centres in the UK (London and Cambridge) and Germany (Wuppertal). A total number of 53 children were included consisting of 26 CD (mean age 12.6 years, 15 female), 11 UC (mean age 11.2 years, 4 female) and 16 healthy controls (age mean 12.1 years, 9 female). Diagnosis of CD and UC was based on standard criteria using clinical, radiological, endoscopic, and histopathological findings in accordance with Porto criteria [13]. As healthy control group, children with macroscopically and histologically normal mucosa as well as no evidence of any underlying GI condition were recruited. Endoscopy was performed by experienced paediatric gastroenterologists, who collected 1–2 additional biopsies within close proximity of the area in which biopsies were taken for routine histology. Biopsies were collected from the duodenum (D; n = 88), terminal ileum (TI; n = 90) and ascending colon (AC; n = 105).

Ethical approval for this study was obtained for all participating hospitals and fully informed written consent was taken from legal guardians and children where appropriate.

### Histological scoring and tissue storage

Biopsies taken for routine histological examination were evaluated by two experienced paediatric histopathologists and the presence of inflammation recorded. Tissue specimens were classified as inflamed if any of the following features were present: significant infiltration of inflammatory cells, ulceration, cryptitis or crypt abscesses. In the absence of these findings, tissue was classified as non-inflamed. Biopsy samples for real-time (RT) analysis were directly transferred into RNAlater (QIAGEN) and stored at −80°C until further processing.

### RNA extraction and reverse transcription PCR

RNA was extracted from biopsies using QIAGEN RNeasy extraction kit according to the manufacturer's instructions. Integrity of extracted RNA was analyzed by agarose gel electrophoresis and quantified using spectrophotometric absorbance at 260 nm. Following, 500 ng of RNA were reverse transcribed using QIAGEN QuantiFast kit, which included DNAse treatment to eliminate any residual genomic DNA.

### Real-Time (RT)-PCR and absolute quantification of mRNA copy numbers

Real Time (RT)-PCR analysis was performed on a Rotor Gene 6000 real time rotary analyzer (Corbett Life Science) using SYBR green methodology. Briefly, reverse transcribed cDNA corresponding to 12.5 ng of RNA were used in a 20 µl PCR reaction containing 10 µl QuantiFast SYBR green master mix (QIAGEN), 2 µl gene specific primers (100 nM of each primer) and 8 µl cDNA in H_2_O. Primer sequences used were as follows: hBD1: forward5′-acc ttc tgc tgt tta ctc tct gct-3′ reverse 5′-gac att gcc ctc cac tgc t-3′ hBD2: forward 5′-cca gcc atc agc cat gag ggt-3′ reverse 5′-gga gcc ctt tct gaa tcc gca-3′ hBD3 forward 5′-agc cta gca gct atg agg atc-3′, reverse 5′-ctt cgg cag cat ttt cgg cca-3′ TNF-alpha forward 5′-ccc agg gac ctc tct cta atc a-3′ reverse 5′-gct aca ggc ttg tca ctc gg-3′ IL-8 forward 5′-atg act tcc aag ctg gcc gtg gc-3′ reverse 5′tct cag ccc tct tca aaa act tc-3′. Cycling conditions were as follows: 10 min 95°C, followed by 40 cycles at 95°C for 20 sec and 60°C for 40 sec. At the end of each run melting point analysis was performed to validate specificity of PCR transcripts. All samples were analyzed in triplicate. Absolute quantification was performed using the standard curve method. Briefly, for each gene tested, reference plasmids containing the target sequence were produced (by cloning into p-drive Vector) and the amount of plasmid measured using a Nano-drop Spectrophotometer (ND-1000, Labtech International). Copy numbers were calculated using Avogadro's formula and standard curves generated by performing serial dilutions of plasmid samples ranging from 10^7^ to 10^2^ copies. In each PCR run a reference standard was included and the primer specific standard curve imported using software provided by Corbett Life Science.

All samples were normalized against the geometric mean of two reference genes (GAPDH and β-actin), which were chosen after testing a group of 7 commonly used reference genes using geNorm Visual Basic application for Microsoft Excel as described previously [17].

### Statistical analysis

Mean expression levels of all genes tested were obtained from triplicate real time PCR measurements. Data are presented as the mean +/− standard error of mean (SEM). Testing for significant differences between groups was performed using an unpaired *t-test* for values with Gaussian distribution and the Mann-Whitney test for values without Gaussian distribution. The Kolmogorov-Smirnov test was utilised to determine Gaussian distribution. For correlation analysis Spearman's rank coefficient was calculated. Values for *P*<0.05 were considered statistically significant. All analysis were performed using Graphpad Prism version 5 (Graphpad Software, San Diego, CA).
